# A fluorescent probe with a vanillin–pyridine–imidazole core structure for carboxylesterase detection in macrophage polarization during bone homeostasis

**DOI:** 10.3389/fchem.2025.1666238

**Published:** 2025-08-21

**Authors:** Hailong Xia, Xianghe Wang, Weichun Huang, Xindong Jiang, Xionggao Han, Chaoyue Wang

**Affiliations:** ^1^ Orthopedics Department, Dongyang People’s Hospital, Jinhua, China; ^2^ The First Affiliated Hospital of Wenzhou Medical University, Wenzhou, China; ^3^ Jinhua Institute of Zhejiang University, Zhejiang University, Jinhua, China; ^4^ Department of Food Science and Nutrition, Hallym University, Chuncheon, Republic of Korea

**Keywords:** carboxylesterase detection, macrophage polarization, fluorescence probe, intracellular imaging, bone homeostasis

## Abstract

In this work, a fluorescent probe, VanPI-CarE, with a vanillin–pyridine–imidazole core structure was developed for carboxylesterase (CarE) detection in macrophage polarization during bone homeostasis. The probe responded to CarE with a distinct fluorescence reporting signal at 490 nm upon excitation at 355 nm. Tests in solution showed the advantages of VanPI-CarE, including high sensitivity, excellent stability under various working conditions, high selectivity, and low cytotoxicity. Further confocal imaging indicated that VanPI-CarE enabled visualization of CarE level in living macrophages. The probe further revealed macrophage polarization in bone homeostasis under both induction and inhibition conditions via CarE detection. These findings provide meaningful insights for inflammation-related research.

## 1 Introduction

Bone homeostasis is an essential balance between bone formation and absorption, which is responsible for the generation, coloboma, and reconstruction of the bone tissues (Tuckermann and Adams, 2021; Zhu et al., 2024). One pathway for generating bone cells is the conversion of immune-related cells after the induction of specific cytokines or agents, while the reverse process—conversion from bone cells back to immune-related cells—also occurs (Mi et al., 2023; Tao et al., 2025). One typical case of conversion is macrophage polarization during bone homeostasis, which is closely associated with inflammation during differentiation to form the classic pro-inflammatory M1 macrophages and the alternative anti-inflammatory M2 macrophages (Bi et al., 2024; Yan et al., 2024). M1 macrophages, which are commonly induced by Toll-like receptors (TLRs) or Th1 cytokines, exhibit strong antigen-presenting capabilities and secrete pro-inflammatory cytokines (Chen et al., 2023; Liu C. et al., 2022). In particular, they are also the precursors of osteoclasts, which produce cytokines to induce bone resorption (Kong et al., 2019). On the other hand, M2 macrophages promote tissue repair through processes such as immune tolerance, tissue remodeling, debris clearance, and immune regulation while also activating tumor angiogenesis, which facilitates tumor growth and immune escape (Chen et al., 2020; Ti et al., 2024). In bone homeostasis, M2 macrophages stimulate differentiation and mineralization by producing osteogenic factors, after which the resulting cells, including mesenchymal stem cells (MSCs), complete bone formation (Daneshvar et al., 2024). Previous investigations revealed that macrophage polarization was closely related to lipid metabolism (Rodriguez et al., 2025). A key factor is oxidized low-density lipoprotein (ox-LDL), which activates macrophage polarization toward the M1 type by affecting TLRs and scavenger receptors (Yang et al., 2022). Meanwhile, inflammation is promoted by cytokines such as interleukin-6 (IL-6) and tumor necrosis factor-*α* (TNF-*α*). For the fatty acids, the saturated species activate the NF-*κ*B signaling pathway to induce M1-type macrophage polarization; on the other hand, the unsaturated species stimulate the peroxisome proliferator-activated receptor (PPAR) pathway to cause anti-inflammatory M2-type macrophage polarization (Li et al., 2023; Mao et al., 2024). Correspondingly, lipid metabolism in macrophage polarization during bone homeostasis has become a research hot spot.

Monitoring lipid metabolism commonly relies on the detection of the blood lipid substances, including total cholesterol (TC), triglycerides (TGs), low-density lipoprotein cholesterol (LDL-C), and high-density lipoprotein cholesterol (HDL-C) (Higashi, 2023; van Wijk et al., 2009). Further molecular and enzymatic indicators are urgently needed to fulfill the necessity of real-time and *in situ* detection. Among the potential candidates, carboxylesterase (CarE) has attracted the attention of researchers due to its significant role in lipid metabolism during endogenous generation, drug digestion, and exogenous toxicant intake (Chen et al., 2018; Liu Y. P. et al., 2024). As a key regulator, CarE mediates the hydrolysis of TGs, which has been studied in liver disorders, including fatty liver, alcoholic hepatitis, and hepatocellular carcinoma (HCC), and obesity-associated inflammatory diseases such as diabetes mellitus (DM) (Dominguez et al., 2014; Hu et al., 2025; Liu Y. et al., 2022). In consideration of its key role in both lipid and carbohydrate metabolism, CarE is a suitable indicator for both lipid-related and inflammation-related events. Therefore, CarE is a potential indicator for macrophage polarization during bone homeostasis. The detection of CarE has been investigated in hepatic and pulmonary cells, while the reports in bone-related induction remain a challenging trial (Cavallero et al., 2024). Accordingly, for CarE detection, the current method is a blood biochemistry test, which requires extracorporeal operation (Merza et al., 2025). The fluorescent probes, with advantageous features including high sensitivity, high specificity, and non-invasive imaging capability, have been introduced for the detection of many molecular indicators (Wang et al., 2024). For CarE detection, in particular, the corresponding fluorescent probes have also been developed to suit the specific application scenarios (Bao et al., 2025; Cui et al., 2024; Li et al., 2025; Liu M. et al., 2025; Ma et al., 2025; Shi et al., 2025; Xing et al., 2025; Xu et al., 2025; Zhang Y. Y. et al., 2025; Zhang Z. M. et al., 2025). One of the most reliable recognition groups for CarE is the carbamate group, which was inspired by inhibitors of acetylcholinesterase and butyrylcholinesterase (Wu et al., 2020). Based on the above information, it is meaningful to develop novel fluorescent probes for CarE detection in macrophage polarization during bone homeostasis. With the introduction of the cooperative indicators, including blood calcium concentration and cytokines (IL-6 and TNF-*α*), the established functioning network might be referable.

**FIGURE 1:**
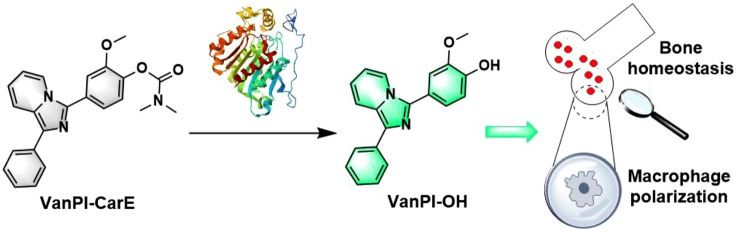
Response of the probe VanPI-CarE to CarE and its detection in macrophage polarization during bone homeostasis.

In this work, after checking the previous investigations, a fluorescent probe with a vanillin–pyridine–imidazole core structure was developed for CarE detection in macrophage polarization during bone homeostasis (Figure 1). The prepared probe, VanPI-CarE, was named based on the subunits of its core structure, including vanillin, pyridine, and imidazole, and its detecting target, CarE. Recently, modification of the fluorophores has been inspired by natural products (Cao et al., 2021; Li et al., 2022; Liu Q. et al., 2024; Shi et al., 2022; Song et al., 2022). Among the reported moieties, vanillin was preferred because of its methoxy group, which serves as an inherent optical auxiliary group (Tang et al., 2012; Yuan et al., 2020). The probe was assembled from the modified fluorophore VanPI-OH, as referenced by Jiang et al. (2025) and Liu Q. Q. et al. (2025), and the reliable carbamate recognition group for CarE (Wu et al., 2020). It was expected to show practical serviceability for the challenging trial of bone homeostasis-related inflammatory regulation, such as macrophage polarization. Tests in solution and imaging in induced macrophages were conducted.

## 2 Experimental procedure

### 2.1 General materials and methods

Commercially available chemicals and enzymes were purchased and used without further purification. In thin-layer chromatography (TLC) and column chromatographic separation, 300–400 mesh silica gel was purchased from Qingdao Haiyang Chemical Co., Ltd. (China). The cell lines used in this study were obtained from the American Type Culture Collection (ATCC) and stored in the central laboratory of the Jinhua Institute of Zhejiang University. The solution system, including phosphate-buffered saline (PBS), was prepared with pure water produced by the Milli-Q Reference Water Purification System (Merck, Darmstadt, Germany). Characterization was performed by nuclear magnetic resonance (NMR) spectroscopy on a Bruker DRX-600 Spectrometer (Germany) and high-resolution mass spectrometry (HRMS) on an AB SCIEX Triple-TOF 4600 System (United States). The UV-VIS spectra tests were conducted on a Shimadzu UV-2550 Spectrophotometer (Shimazu, Kyoto, Japan), while the fluorescence signals were measured on a Hitachi F-7000 Fluorescence Spectrophotometer (Japan). The confocal imaging experiments were performed on a Leica Mai Tai SP8 Microscope (Germany).

The probe VanPI-CarE was stored as a 1 mM stock solution, with dimethyl sulfoxide (DMSO) as the solvent. The solution system at a total volume of 200 µL for the detection consisted of 20 µL DMSO (containing the probe), 80 µL PBS (final concentration 10 mM, for preparing CarE), and 100 µL pure water (containing the aqueous analytes). Unless the condition was being tested, the working conditions were set as pH 7.4, incubation time 20 min, incubation temperature 37 °C, photomultiplier voltage 600 V, excitation/emission slit width 5 nm * 5 nm, and excitation wavelength 355 nm. The signal collection range in the confocal imaging experiments was 450 nm–600 nm in the green channel.

### 2.2 Synthesis of compounds

**FIGURE 2:**
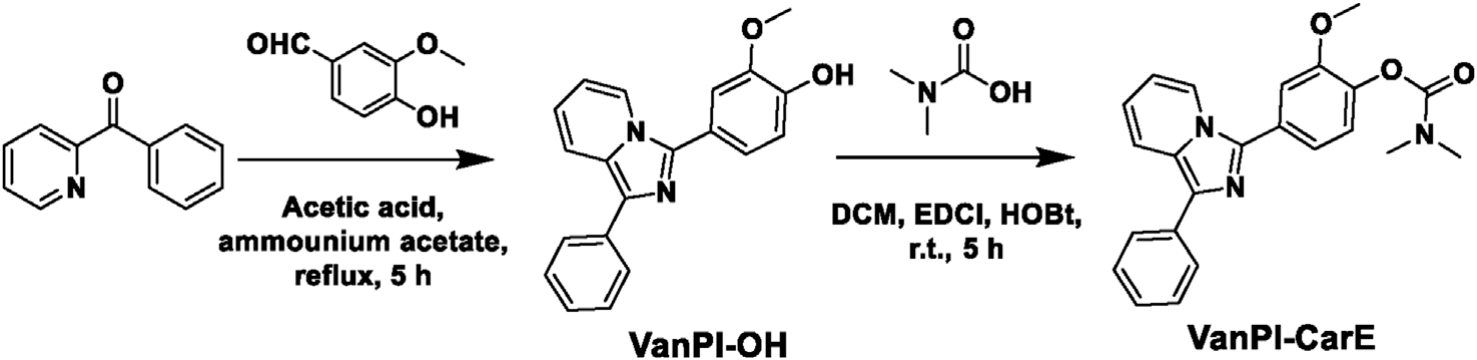
General synthetic route of the probe VanPI-CarE. DCM, dichloromethane; EDCI, 1-ethyl-3-(3-dimethylaminopropyl) carbodiimide hydrochloride; HOBt, 1-hydroxybenzotriazole; r.t., room temperature.

The chemical synthesis process of the probe VanPI-CarE is depicted in Figure 2. There were two main steps. At first, 15 mL of acetic acid was added to a 50-mL round-bottom flask to dissolve the reagents phenyl(pyridin-2-yl)methanone (0.27 g, 1.5 mmol), ammonium acetate (0.15 g, 2 mmol), and vanillin (0.23 g, 1.5 mmol). The reaction was carried out under reflux for 5 h, and its completion was monitored by TLC. Subsequently, ice water was added to the reaction system, and the precipitate was collected. After column chromatography (petroleum ether: ethyl acetate = 5:1), the fluorophore VanPI-OH was acquired as a yellow solid (yield 75.2%). The ^1^H NMR spectrum (600 MHz, CDCl_3_) showed signals at *δ* 9.79 (s, 1H), 8.18 (d, *J* = 7.3 Hz, 1H), 7.92 (d, *J* = 7.2 Hz, 2H), 7.81 (d, *J* = 9.3 Hz, 1H), 7.45 (t, *J* = 7.7 Hz, 2H), 7.35 (d, *J* = 1.8 Hz, 1H), 7.29 (t, *J* = 7.4 Hz, 1H), 7.28–7.24 (m, 1H), 7.01 (d, *J* = 8.0 Hz, 1H), 6.76 (dd, *J* = 9.7, 6.3 Hz, 1H), 6.55 (t, *J* = 7.1 Hz, 1H), and 3.91 (s, 3H). The ^13^C NMR spectrum (151 MHz, CDCl_3_) showed peaks at *δ* 147.35, 146.66, 138.27, 134.73, 131.40, 128.73, 127.33, 126.86, 126.56, 121.87, 120.91, 119.62, 119.03, 114.68. 114.53, 113.20, 111.90, and 56.08. HRMS (Q-TOF m/z) provided a calculated value of 317.1290 for [C_20_H_17_N_2_O_2_]^+^ and a found value of 317.1279.

Furthermore, 20 mL of dichloromethane (DCM) was added to a 50-mL round-bottom flask to dissolve VanPI-OH (0.5 mmol), *N*,*N*-dimethyl carbamic acid (0.5 mmol), 1-ethyl-3-(3-dimethylaminopropyl) carbodiimide hydrochloride (EDCI, 1 mmol), and 1-hydroxybenzotriazole (HOBt, 0.5 mmol). The reaction was carried out at room temperature for 5 h, and its completion was monitored by TLC. The solvent was vaporized, and following that, column chromatography (petroleum ether: ethyl acetate = 16:1) was conducted. The probe VanPI-CarE was acquired as a yellow solid (yield 48.6%). The ^1^H NMR spectrum (600 MHz, CDCl_3_) showed signals at *δ* 8.27 (d, *J* = 7.3 Hz, 1H), 7.96 (d, *J* = 7.3 Hz, 2H), 7.85 (d, *J* = 9.2 Hz, 1H), 7.50–7.48 (m, 3H), 7.38 (dd, *J* = 8.2, 1.9 Hz, 1H), 7.32 (t, *J* = 7.4 Hz, 1H), 7.26 (d, *J* = 8.1 Hz, 1H), 6.80 (dd, *J* = 9.2, 6.3 Hz, 1H), 6.59 (t, *J* = 6.5 Hz, 1H), 3.95 (s, 3H), 3.18 (s, 3H), and 3.06 (s, 3H). The ^13^C NMR spectrum (151 MHz, CDCl_3_) showed peaks at *δ* 154.57, 152.39, 141.16, 137.67, 134.86, 131.87, 128.75, 128.26, 127.73, 126.86, 126.61, 123.65, 121.92, 119.97, 119.81, 119.11, 113.35, 113.26, 56.28, 36.86, and 36.65. HRMS (Q-TOF m/z) provided a calculated value of 388.1661 for [C_23_H_22_N_3_O_3_]^+^ and a found value of 388.1643.

### 2.3 Determination of fluorescence quantum yields

The reference method using an ethanol solution of rhodamine B (10 μM, Φ = 0.69, λ_ex_ = 365 nm) was employed to calculate the fluorescence quantum yield (FQY) values. In this study, the FQY values of the probe VanPI-CarE and the detection product VanPI-OH were 0.15 and 0.71, respectively.

### 2.4 Determination of the limit of detection

In this work, the limit of detection (LOD) was calculated using the formula LOD = 3σ/k, where the background noise σ was obtained from 25 independent tests of the solution system containing only the probe and the slope k was determined from the linear regression equation. Thus, σ = 0.4985, k = 88.52, and LOD = 0.017 U/mL.

### 2.5 Determination of cell viability and confocal imaging

Cell viability was tested on RAW264.7 (mouse monocyte macrophage leukemia cells) and MC3T3-E1 (mouse embryonic osteoblast precursor cells) using the thiazolyl blue (MTT) assay (Buranaamnuay, 2021). Absorbance at 570 nm was measured.

Moreover, RAW264.7 cells were incubated in Dulbecco’s modified Eagle’s medium (DMEM) with hydroxyethyl piperazine ethane sulfonic acid buffer (HEPES, pH 7.2), 10% fetal bovine serum (FBS), and 1% penicillin–streptomycin at 37 °C under a 5% CO_2_ atmosphere. HEPES was added to improve the solubility of receptor activator of nuclear factor-κB ligand (RANKL). The RAW264.7 cell line was chosen for its relevance to the study of macrophage polarization in bone homeostasis. The cells were divided into five groups. The first group served as the original condition, incubated with HEPES for 30 min, followed by incubation with the probe VanPI-CarE (10 μM) for 30 min, and then imaged. The second group served as the inhibited condition, incubated with the CarE inhibitor bis(4-nitrophenyl)phosphate (BNPP) at 1 μM for 30 min, followed by incubation with VanPI-CarE (10 μM) for 30 min, and then imaged. The third group served as the stimulated condition, which was pre-treated with oxidized low-density lipoprotein (ox-LDL) at 20 μg/mL during the last 12 h of culturing to induce the CarE level, incubated with VanPI-CarE (10 μM) for 30 min, and then imaged. The fourth group served as the bone homeostasis-related macrophage polarization condition, which was induced by RANKL (100 ng/mL) during the last 12 h of culturing (Elango et al., 2021), incubated with HEPES for 30 min, followed by incubation with VanPI-CarE (10 μM) for 30 min, and then imaged. The fifth group served as the macrophage polarization-inhibited condition, which was induced by RANKL, treated with the RANKL inhibitor denosumab (1 μg/mL) during the last 1 h of culturing (Deligiorgi and Trafalis, 2021), incubated with HEPES for 30 min, followed by incubation with VanPI-CarE (10 μM) for 30 min, and then imaged. The fluorescence signals in the green channel of 450 nm–600 nm were collected when the excitation wavelength was set to 355 nm.

## 3 Results and discussion

### 3.1 Chemical synthesis of VanPI-CarE

The general synthetic route of the probe VanPI-CarE is depicted in Figure 2 as comprising two main steps. The initial step was the formation of the fluorophore VanPI-CarE from the cyclization of vanillin, phenyl(pyridin-2-yl)methanone, and ammonium acetate (Shi et al., 2025; Yuan et al., 2020). The vanillin–pyridine–imidazole core structure was constructed thereby. The second step was anchoring the carbamate recognition onto the fluorophore to yield the probe VanPI-CarE. The structures of the probe and the fluorophore were confirmed by satisfactory characterization data (^1^H NMR, ^13^C NMR, and HRMS, Supplementary Figures S1–S6 in Supporting Information). The recognition mechanism was supported by previous reports and variations in the HRMS data.

### 3.2 Optical performance in solution system

**FIGURE 3:**
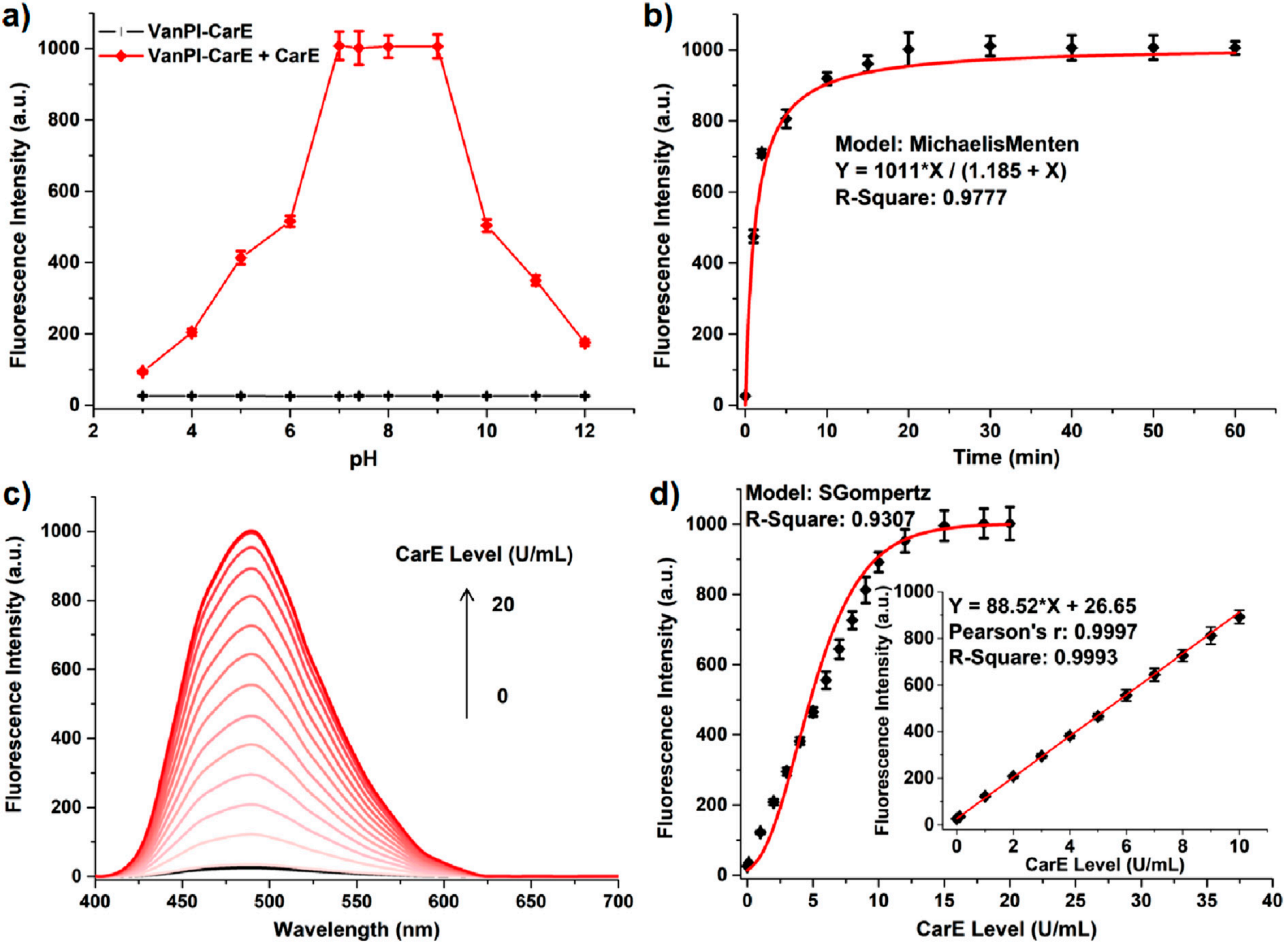
**(a)** Fluorescence intensity at 490 nm of VanPI-CarE (10 µM) in the absence and presence of CarE (20 U/mL) in various pH conditions (3.0–12.0). **(b)** Fluorescence intensity at 490 nm of VanPI-CarE (10 µM) with CarE (20 U/mL) in various incubation time conditions (0 min–60 min). **(c)** Fluorescence spectra of VanPI-CarE (10 µM) with various CarE levels (0 U/mL–20 U/mL). **(d)** Correlation between the CarE level (0 U/mL–20 U/mL) and the fluorescence intensity at 490 nm. Inner: linear correlation range (0 U/mL–10 U/mL). Conditions (unless being tested): pH 7.4, 37 °C, 20 min, 5 nm * 5 nm, 600 V, and λ_ex_ = 355 nm.

When the optical performance was studied, the UV–VIS absorption and fluorescence spectra were examined to provide the initial information. In this work, the probe VanPI-CarE (10 μM) exhibited a visible peak at 530 nm (due to the frequency-doubling effect), while recognition with CarE (20 U/mL) for 20 min at 37 °C resulted in a decrease in the signal (Supplementary Figures S7a). More importantly, for the aspect of the fluorescence reporting signal, when the excitation wavelength was set to 355 nm, the probe VanPI-CarE (10 μM) was almost non-fluorescent, while recognition with CarE (20 U/mL) for 20 min at 37 °C caused a remarkable enhancement in the peak at 490 nm (Supplementary Figures S7b). The response scale referred an over 35-fold fluorescence enhancement, which was suitable for establishing the system of turning-on recognition. Based on the collection of both the absorbance and fluorescence data, the FQY values of the probe VanPI-CarE and the detection product VanPI-OH were 0.15 and 0.71, respectively. Since the basic signal variation during CarE recognition had been studied, the following experiments were carried out to examine the working conditions, including pH, incubation time, and temperature. Recognition of the enzymatic indicator is commonly affected by the pH condition. In this study, the probe VanPI-CarE showed no obvious fluorescence signal within the whole tested range of 3.0–12.0, while the fluorescence reporting signal with a certain intensity after recognition between VanPI-CarE and CarE remained stable in the range of 7.0–9.0 (Figure 3a). This result indicated the considerable potential for detection in physiological and pathological procedures. Meanwhile, since the recognition time is usually a significant factor, it was also tested by setting different checking points. Recognition of VanPI-CarE toward CarE was completed within 20 min, which is a shorter period than that in similar reports (Figure 3b). The time-dependent response followed the Michaelis–Menten model, with parameters including V_max_ = 1,011 min^−1^ and K_m_ = 1.185 U/mL, consistent with typical values of carboxylesterase (Nagaoka et al., 2024). For the condition of incubation temperature, VanPI-CarE itself showed no obvious fluorescence signal in the tested range of 25 °C–45 °C, while the fluorescence reporting signal remained stable in the range of 35 °C–40 °C (Supplementary Figures S8). This result was also consistent with the requirements of the physiological micro-environment.

After the working conditions were investigated, the correlation between the reporting signal intensity at 490 nm and the CarE level (0–20 U/mL) in the solution system containing VanPI-CarE (10 μM) was established. The upper limit of the CarE level was set at 20 U/mL because this level ensured a relatively transparent solution and fulfilled the requirements for physiological detection. As the CarE level increased gradually, the fluorescence reporting signal correspondingly enhanced, reaching a saturated value at a CarE level of 15 U/mL (Figures 3c, d). A linear correlation was found in the range of 0 U/mL–10 U/mL, with a Pearson’s r value of 0.9997 (Figure 3d Inner). Using the formula 3σ/k, the LOD value was determined to be 0.017 U/mL, indicating relatively high sensitivity. Both the linear range and the LOD value are suitable for the potential research scenarios in this work. Therefore, in the solution system, the probe VanPI-CarE showed potential optical capabilities for CarE detection.

### 3.3 Selectivity toward CarE

**FIGURE 4:**
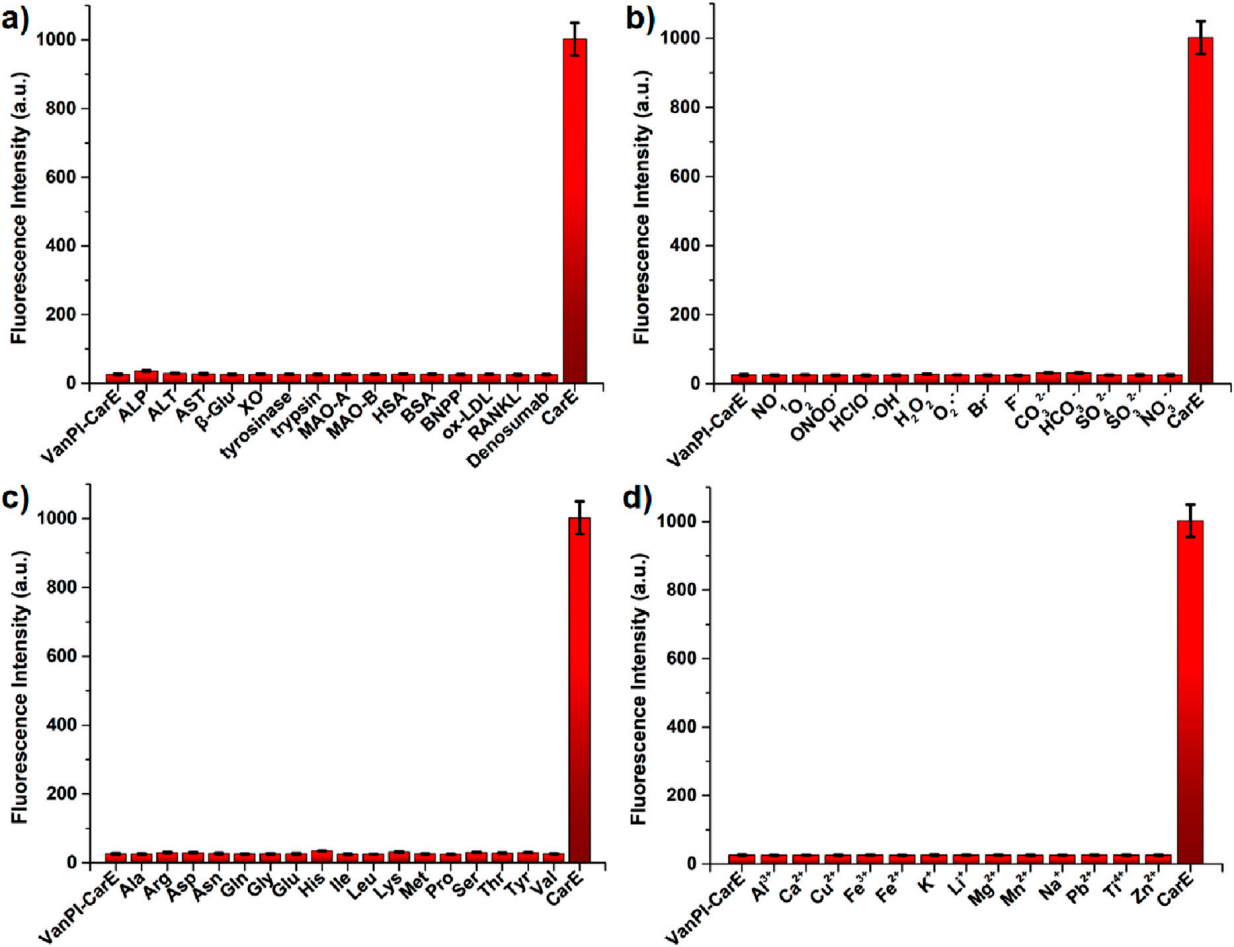
Fluorescence intensity at 490 nm of VanPI-CarE (10 µM) with various competing species: **(a)** enzymes and incubation agents: ALP (20 U/mL), ALT (20 U/mL), AST (20 U/mL), *β*-Glu (20 U/mL), XO (20 mU/mL), tyrosinase (20 U/mL), trypsin (20 mg/mL), MAO-A (20 mg/mL), MAO-B (20 mg/mL), HSA (10 mM), BSA (10 mM), BNPP (1 μM), ox-LDL (20 μg/mL), RANKL (100 ng/mL), denosumab (1 μg/mL), and CarE (20 U/mL); **(b)** ROS/RNS and anions; **(c)** amino acids; **(d)** cations. Conditions: other analytes: 1 mM; pH 7.4, 37 °C, 20 min, 5 nm * 5 nm, 600 V, and λ_ex_ = 355 nm. ALP, alkaline phosphatase; ALT, alanine aminotransferase; AST, aspartate aminotransferase; *β*-Glu, *β*-glucosidases; XO, xanthine oxidase; MAO-A, monoamine oxidase-A; MAO-B, monoamine oxidase-B; HSA, human serum albumin; BSA, bovine serum albumin; BNPP, bis(4-nitrophenyl)phosphate; ox-LDL, oxidized low-density lipoprotein; RANKL, receptor activator of nuclear factor-κB ligand.

In the next step, the selectivity of the probe VanPI-CarE (10 µM) toward CarE (20 U/mL) was investigated. The most concerned species were the competing enzymes, including alkaline phosphatase (ALP), alanine aminotransferase (ALT), aspartate aminotransferase (AST), *β*-glucosidases (*β*-Glu), xanthine oxidase (XO), tyrosinase, trypsin, monoamine oxidase-A (MAO-A), monoamine oxidase-B (MAO-B), human serum albumin (HSA), and bovine serum albumin (BSA) from the similar physiological micro-environment of CarE (Figure 4a). In particular, the inhibition and induction agents in intracellular imaging, including BNPP, ox-LDL, RANKL, and denosumab, were involved. None of the tested species, except CarE, led to a remarkable enhancement of the fluorescence reporting signal. In consideration of their activity in physiological events, the reactive oxygen/nitrogen species (ROS/RNS), including NO, ^1^O_2_, ONOO^−^, HClO, OH, H_2_O_2_, and O_2_^−^, and anions, including Br^−^, F^−^, CO_3_^2−^, HCO_3_^−^, SO_4_^2−^, SO_3_^2−^, and NO_3_^−^, were also tested in this section (Figure 4b). None of them caused any detectable fluorescence reporting signal. In further steps, the tests covered more analytes, including the usual amino acids (Ala, Arg, Asp, Asn, Gln, Gly, Glu, His, Ile, Leu, Lys, Met, Pro, Ser, Thr, Tyr, and Val; Figure 4c) and cations (Al^3+^, Ca^2+^, Cu^2+^, Fe^3+^, Fe^2+^, K^+^, Li^+^, Mg^2+^, Mn^2+^, Na^+^, Pb^2+^, Ti^4+^, and Zn^2+^; Figure 4d). None of the tested analytes produced a notable fluorescence reporting signal. Therefore, in the solution system, the high selectivity of the probe VanPI-CarE toward CarE was guaranteed.

### 3.4 Intracellular imaging for CarE

This work focused on carboxylesterase detection in macrophage polarization during bone homeostasis. Thus, bone homeostasis-related macrophages and precursor cells, including the RAW264.7 (Supplementary Figures S9a) and MC3T3-E1 (Supplementary Figures S9a) cell lines, were tested for cell viability using a standard MTT assay. After 24 h of incubation, both the cell lines retained over 90% cell viability when the working concentration of the probe gradually increased to 50 µM. Thus, VanPI-CarE inferred low cytotoxicity for imaging in living macrophages.

**FIGURE 5:**
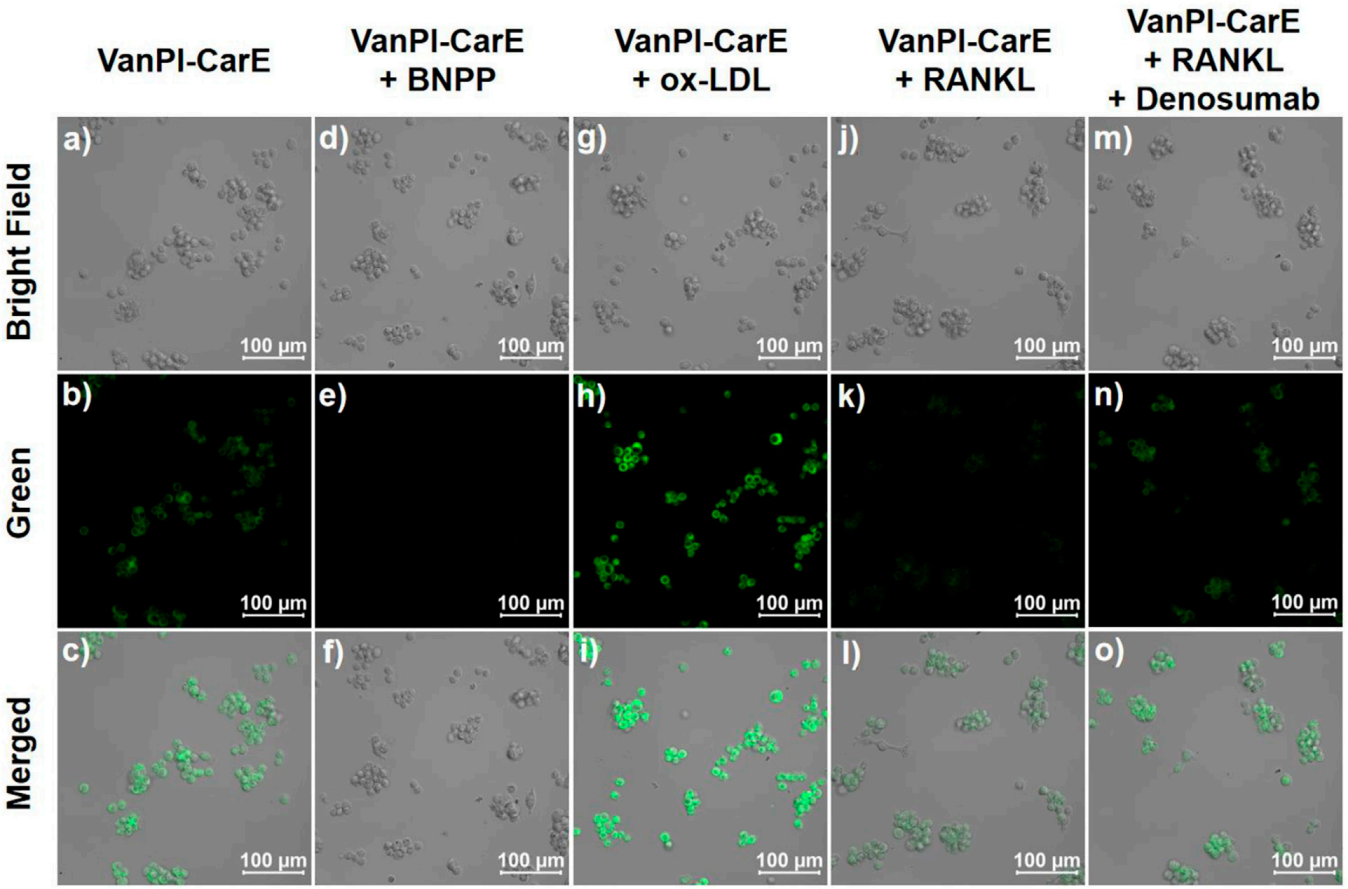
Confocal images of RAW264.7 cells in various conditions before being imaged. **(a–c)** Incubation with HEPES for 30 min, followed by incubation with VanPI-CarE (10 μM) for 30 min. **(d–f)** Incubation with BNPP (1 μM) for 30 min, followed by incubation with VanPI-CarE (10 μM) for 30 min. **(g–i)** Pre-treated with ox-LDL (20 μg/mL) during the last 12 h of culturing, followed by incubation with VanPI-CarE (10 μM) for 30 min; **(j–l)** pre-treated with RANKL (100 ng/mL) during the last 12 h of culturing, followed by incubation with VanPI-CarE (10 μM) for 30 min; **(m–o)** pre-treated with RANKL (100 ng/mL) during the last 12 h of culturing and denosumab (1 μg/mL) during the last 1 h of culturing and incubated with VanPI-CarE (10 μM) for 30 min. Conditions: pH 7.4, 37 °C, λ_ex_ = 355 nm, green channel 450 nm–600 nm, and scale bar = 100 µm. BNPP: bis(4-nitrophenyl)phosphate; ox-LDL: oxidized low-density lipoprotein; RANKL: receptor activator of nuclear factor-κB ligand.

RAW264.7 cells were maintained in an uninduced state before the confocal experiments because bone homeostasis-related macrophage polarization requires induction during culturing. The cells were divided into five groups according to the different treatment conditions. The first group, which represented the original condition, was incubated with HEPES for 30 min, followed by incubation with the probe VanPI-CarE (10 μM) for 30 min, and then imaged (Figures 5a–c). Since the living RAW264.7 cells bore a certain level of CarE, the fluorescence reporting signal was observed in the green channel. In the second group, in which CarE was inhibited by BNPP (1 μM), the following incubation with VanPI-CarE resulted in a remarkable decrease in the fluorescence reporting signal (Figures 5d–f). On the contrary, the third group was pre-treated with ox-LDL (20 μg/mL) during the last 12 h of culturing before being incubated with VanPI-CarE for 30 min and then imaged (Figures 5g–i). In this group, the CarE level was stimulated, and the fluorescence reporting signal was notably enhanced. The results from the initial three groups suggested that VanPI-CarE was capable of visualizing the CarE levels in living macrophages under both inhibition and activation conditions. Then, the following two groups were associated with macrophage polarization during bone homeostasis. The fourth group, which served as the bone homeostasis-related macrophage polarization condition, was induced by RANKL (100 ng/mL) during the last 12 h of culturing before being incubated with HEPES for 30 min, followed by incubation with VanPI-CarE (10 μM) for 30 min, and then imaged (Figures 5j–l). Correspondingly, the fluorescence reporting signal in the green channel exhibited a remarkable decrease, which was consistent with the fact that the M1-type macrophage polarization process induced by ox-LDL caused inflammation and affected the metabolism of fatty acids (Yang et al., 2022). Finally, the fifth group was established on the basis of the fourth condition. After induction with RANKL, the cells were treated with the RANKL inhibitor denosumab (1 μg/mL) during the last 1 h of culturing before incubation with HEPES and VanPI-CarE (Figures 5m–o). The fluorescence reporting signal subsequently indicated a recovery close to the original uninduced condition in the first group, suggesting a corresponding restoration of the CarE level. Therefore, VanPI-CarE achieved reflection of macrophage polarization in bone homeostasis, regardless of induction or inhibition, by visualizing the CarE level.

## 4 Conclusion

In conclusion, on the basis of the investigation of previous research, a fluorescent probe with a vanillin–pyridine–imidazole core structure was developed for CarE detection in macrophage polarization during bone homeostasis. The acquired probe VanPI-CarE exhibited an obvious fluorescence reporting signal at 490 nm upon excitation at 355 nm. In the solution system, the FQY values of the probe VanPI-CarE and the detection product VanPI-OH were 0.15 and 0.71, respectively. After the investigation of the working conditions, including pH, incubation time, and temperature, the results indicated that the fluorescence reporting signal reached a saturated value within 20 min and remained stable at pH 7.0–9.0 and 35 °C–40 °C. The optical performance was beneficial for fulfilling the requirements of the physiological micro-environment. The intensity of the fluorescence reporting signal at 490 nm exhibited a dose-dependent enhancement along with an increase in the CarE level. A linear correlation was found in the range of 0 U/mL–10 U/mL, and the LOD value was determined to be 0.017 U/mL, which suggested relatively high sensitivity. VanPI-CarE also showed high selectivity toward CarE over competing species and low cytotoxicity toward bone homeostasis-related macrophages and precursor cells. Moreover, the confocal imaging results suggested that VanPI-CarE successfully visualized the CarE level in living macrophages under both inhibition and activation conditions. Furthermore, in macrophage polarization during bone homeostasis, both the induction and inhibition conditions were reflected by VanPI-CarE via CarE detection. Future research should focus on the enhancement of the fluorescence reporting signal intensity in imaging due to the relatively short emission wavelength. The possible solutions might include the precise modification of the substitutes and enrichment with functional carriers (Ding and Ren, 2023; Dou et al., 2021). This work provides valuable information on carboxylesterase detection in macrophage polarization during bone homeostasis, which is relevant for inflammation-related research.

## Data Availability

The datasets presented in this study can be found in online repositories. The names of the repository/repositories and accession number(s) can be found in the article/Supplementary Material.

## References

[B1] Bao H. L. Xu X. B. Xia Z. L. Wang C. Y. (2025). A fluorescent probe derived from triphenylamine-benzofuranone for monitoring carboxylesterases and its imaging in drug-induced liver cellular injury. J. Photoch. Photobio. A 459, 116005. 10.1016/j.jphotochem.2024.116005

[B2] Bi Z. G. Cai Y. M. Shi X. T. Chen J. T. Li D. S. Zhang P. B. (2024). Macrophage-mediated immunomodulation in biomaterial-assisted bone repair: molecular insights and therapeutic prospects. Chem. Eng. J. 488, 150631. 10.1016/j.cej.2024.150631

[B3] Buranaamnuay K. (2021). The MTT assay application to measure the viability of spermatozoa: a variety of the assay protocols. Open Vet. J. 11, 251–269. 10.5455/ovj.2021.v11.i2.9 34307082 PMC8288735

[B4] Cao R. Y. Zhang Y. Feng Z. Liu S. Y. Liu Y. F. Zheng H. C. (2021). The effective role of natural product berberine in modulating oxidative stress and inflammation related atherosclerosis: novel insights into the gut-heart axis evidenced by genetic sequencing analysis. Front. Pharnacol. 12, 764994. 10.3389/fphar.2021.764994 PMC8727899 35002703

[B5] Cavallero A. Donadel G. Puccini P. Gervasi P. G. Gabisonia K. Longo V. (2024). New insight on porcine carboxylesterases expression and activity in lung tissues. Res. Vet. Sci. 175, 105314. 10.1016/j.rvsc.2024.105314 38823354

[B6] Chen F. Zhang B. Parker R. B. Laizure S. C. (2018). Clinical implications of genetic variation in carboxylesterase drug metabolism. Expert Opin. Drug Met. 14, 131–142. 10.1080/17425255.2018.1420164 29264996

[B7] Chen Y. N. Hu M. R. Wang L. Chen W. D. (2020). Macrophage M1/M2 polarization. Eur. J. Pharmacol. 877, 173090. 10.1016/j.ejphar.2020.173090 32234529

[B8] Chen S. Z. Saeed A. F. U. H. Liu Q. Jiang Q. Xu H. Z. Xiao G. G. (2023). Macrophages in immunoregulation and therapeutics. Signal Transduct. Tar. 8, 207. 10.1038/s41392-023-01452-1 PMC10200802 37211559

[B9] Cui Z. H. Wang Y. F. Wang G. Feng B. D. Lewis S. E. Wang K. (2024). Amphiphilic azulene-based fluorescent probe for simultaneous monitoring of fluctuations in carboxylesterase activity in diverse biological samples from a single organism. Anal. Chem. 96, 19732–19739. 10.1021/acs.analchem.4c04926 39587379 PMC11656413

[B10] Daneshvar A. Nemati P. Azadi A. Amid R. Kadkhodazadeh M. (2024). M2 macrophage-derived exosomes for bone regeneration: a systematic review. Arch. Oral Biol. 166, 106034. 10.1016/j.archoralbio.2024.106034 38943857

[B11] Deligiorgi M. V. Trafalis D. T. (2021). The safety profile of denosumab in oncology beyond the safety of denosumab as an anti-osteoporotic agent: still more to learn. Expert Opin. Drug Saf. 20, 191–213. 10.1080/14740338.2021.1861246 33287586

[B12] Ding C. P. Ren T. B. (2023). Near infrared fluorescent probes for detecting and imaging active small molecules. Coordin. Chem. Rev. 482, 215080. 10.1016/j.ccr.2023.215080

[B13] Dominguez E. Galmozzi A. Chang J. W. Hsu K. L. Pawlak J. Li W. W. (2014). Integrated phenotypic and activity-based profiling links Ces3 to obesity and diabetes. Nat. Chem. Biol. 10, 113–121. 10.1038/nchembio.1429 24362705 PMC3953460

[B14] Dou X. L. Sun K. Chen H. B. Jiang Y. F. Wu L. Mei J. (2021). Nanoscale metal-organic frameworks as fluorescence sensors for food safety. Antibiotics-Basel 10, 358. 10.3390/antibiotics10040358 33800674 PMC8067089

[B15] Elango J. Bao B. Wu W. H. (2021). The hidden secrets of soluble RANKL in bone biology. Cytokine 144, 155559. 10.1016/j.cyto.2021.155559 33994070

[B16] Higashi Y. (2023). Endothelial function in dyslipidemia: roles of LDL-Cholesterol, HDL-cholesterol and triglycerides. Cells-Basel 12, 1293. 10.3390/cells12091293 PMC10177132 37174693

[B17] Hu S. J. Liu X. Ding Y. T. Chen J. Wang X. W. (2025). Effects of exercise and walnut oil on CES1 and inflammatory factors in the liver of type 2 diabetic rats. Eur. J. Med. Res. 30, 128. 10.1186/s40001-025-02377-x 39994797 PMC11849220

[B18] Jiang X. D. Lin B. M. Xia H. L. Zhang J. Huang W. C. Wang C. Y. (2025). An isovanillin-derived fluorescent probe with imidazo-pyridin for monitoring cysteine level in macrophage inflammatory regulation of bone homeostasis. J. Photoch. Photobio. A 462, 116186. 10.1016/j.jphotochem.2024.116186

[B19] Kong L. B. Wang Y. H. Smith W. Hao D. J. (2019). Macrophages in bone homeostasis. Curr. Stem Cell Res. Ther. 14, 474–481. 10.2174/1574888x14666190214163815 30767753

[B20] Li Y. Cheng S. H. Tian Y. Zhang Y. N. Zhao Y. (2022). Recent ring distortion reactions for diversifying complex natural products. Nat. Prod. Rep. 39, 1970–1992. 10.1039/d2np00027j 35972343

[B21] Li M. Y. Wu Y. Z. Qiu J. G. Lei J. X. Li M. X. Xu N. (2023). Huangqin decoction ameliorates ulcerative colitis by regulating fatty acid metabolism to mediate macrophage polarization via activating FFAR4-AMPK-PPARα pathway. J. Ethnopharmacol. 311, 116430. 10.1016/j.jep.2023.116430 36997133

[B22] Li X. Y. Meng Z. Y. Gong S. Liang Y. Y. Zhang Y. Xu X. (2025). Synthesis of a new camphor-derived carboxylesterase-activated fluorescent probe for sensitive detection of dimethoate residues in agricultural products and its applications in biological systems. Food Chem. 464, 141625. 10.1016/j.foodchem.2024.141625 39426261

[B23] Liu C. Hu F. Q. Jiao G. L. Guo Y. Zhou P. Zhang Y. N. (2022a). Dental pulp stem cell-derived exosomes suppress M1 macrophage polarization through the ROS-MAPK-NFκB P65 signaling pathway after spinal cord injury. J. Nanobiotechnol. 20, 65. 10.1186/s12951-022-01273-4 PMC8811988 35109874

[B24] Liu Y. He Z. X. Yang Y. T. Li X. H. Li Z. F. Ma H. M. (2022b). New fluorescent probe with recognition moiety of bipiperidinyl reveals the rise of hepatocellular carboxylesterase activity during heat shock. Biosens. Bioelectron. 211, 114392. 10.1016/j.bios.2022.114392 35609457

[B25] Liu Y. P. Li J. P. Zhu H. J. (2024a). Regulation of carboxylesterases and its impact on pharmacokinetics and pharmacodynamics: an up-to-date review. Expert Opin. Drug Met. 20, 377–397. 10.1080/17425255.2024.2348491 PMC11151177 38706437

[B26] Liu Q. Chen X. H. Tan Y. R. Liu J. Zhu M. Y. Li D. L. (2024b). Natural products as glycolytic inhibitors for cervical cancer treatment: a comprehensive review. Biomed. Pharmacother. 175, 116708. 10.1016/j.biopha.2024.116708 38723515

[B27] Liu M. Ding C. X. Yi Q. Y. Quan H. Y. Li W. J. Wang Y. P. (2025a). Carboxylesterase-activated hepatocyte-targeting fluorescent probe for drug-induced liver injury diagnosis. Bioorg. Chem. 162, 108587. 10.1016/j.bioorg.2025.108587 40381462

[B28] Liu Q. Q. Zhu Z. Q. Lv H. Y. Huang B. Y. (2025b). Developing a vanillin-derived imidazo-pyridin-containing fluorescent probe for imaging cysteine in living pulmonary cells under oxygen supply variation. Spectrochim. Acta A 325, 125107. 10.1016/j.saa.2024.125107 39260242

[B29] Ma M. Zhang S. Q. Li J. K. Zhao L. H. Song D. Q. Ma P. Y. (2025). Carboxylesterase 2-based fluorescent probe with large stokes shift for differentiating colitis from bowel cancer. Sens. Actuat. B-Chem. 426, 137057. 10.1016/j.snb.2024.137057

[B30] Mao N. N. Yu Y. M. Lu X. Q. Yang Y. Liu Z. G. Wang D. Y. (2024). Preventive effects of matrine on LPS-induced inflammation in RAW 264.7 cells and intestinal damage in mice through the TLR4/NF-κB/MAPK pathway. Int. Immunopharmacol. 143, 113432. 10.1016/j.intimp.2024.113432 39447411

[B31] Merza W. M. Yaseen A. K. Mahmood M. A. (2025). FSH, LH, lipid and adipokines in polycystic ovary syndrome: clinical biochemistry insights for diagnosis and management. J. Steroid. Biochem. 251, 106773. 10.1016/j.jsbmb.2025.106773 40334996

[B32] Mi B. B. Xiong Y. Zha K. K. Cao F. Q. Zhou W. Abbaszadeh S. (2023). Immune homeostasis modulation by hydrogel-guided delivery systems: a tool for accelerated bone regeneration. Biomater. Sci.-UK 11, 6035–6059. 10.1039/d3bm00544e 37522328

[B33] Nagaoka M. Sakai Y. Nakajima M. Fukami T. (2024). Role of carboxylesterase and arylacetamide deacetylase in drug metabolism, physiology, and pathology. Biochem. Pharmacol. 223, 116128. 10.1016/j.bcp.2024.116128 38492781

[B34] Rodriguez J. P. Casas J. Balboa M. A. Balsinde J. (2025). Bioactive lipid signaling and lipidomics in macrophage polarization: impact on inflammation and immune regulation. Front. Immunol. 16, 1550500. 10.3389/fimmu.2025.1550500 40028333 PMC11867965

[B35] Shi J. W. Li T. T. Dong J. Wu Y. Y. Wang W. R. Wang C. N. (2022). Neurotoxicity and structure-activity relationships of resveratrol and its two natural analogs, 4,4′-Dihydroxystilbene and pinosylvin. Nat. Prod. Commun. 17, 1934578X221113707. 10.1177/1934578x221113707

[B36] Shi J. Y. Wang C. Y. Xia Y. F. Guo M. Y. Yang W. Yang Y. S. (2025). A fluorescent probe for detecting carboxylesterase level on investigating Chinese medicine water decoction efficacy. Spectrochim. Acta A 338, 126187. 10.1016/j.saa.2025.126187 40215850

[B37] Song K. N. Li M. C. Yang Y. Q. Zhang Z. Zhu Q. Liu J. Y. (2022). Natural flavonolignans as potential therapeutic agents against common diseases. J. Pharm. Pharmacol. 74, 337–350. 10.1093/jpp/rgab159 34923582

[B38] Tang J. F. Lv X. H. Wang X. L. Sun J. Zhang Y. B. Yang Y. S. (2012). Design, synthesis, biological evaluation and molecular modeling of novel 1,3,4-oxadiazole derivatives based on vanillic acid as potential immunosuppressive agents. Bioorgan. Med. Chem. 20, 4226–4236. 10.1016/j.bmc.2012.05.055 22727369

[B39] Tao R. Y. Liu C. R. Wong P. Y. Huang T. Alt V. Rupp M. (2025). Advances in immune mechanisms and developing immune-targeted therapies for osteoporosis: a systematic review. Pharmacol. Res. 218, 107835. 10.1016/j.phrs.2025.107835 40550406

[B40] Ti D. D. Yi J. Chen H. H. Hao H. J. Shi C. M. (2024). The role of mesenchymal stem/stromal cells secretome in macrophage polarization: perspectives on treating inflammatory diseases. Curr. Stem Cell Res. T. 19, 894–905. 10.2174/1574888x18666230811093101 37723965

[B41] Tuckermann J. Adams R. H. (2021). The endothelium-bone axis in development, homeostasis and bone and joint disease. Nat. Rev. Rheumatol. 17, 608–620. 10.1038/s41584-021-00682-3 34480164 PMC7612477

[B42] van Wijk D. F. Stroes E. S. G. Kastelein J. J. P. (2009). Lipid measures and cardiovascular disease prediction. Dis. Markers 26, 209–216. 10.1155/2009/143680 19773610 PMC3833241

[B43] Wang X. Ding Q. Groleau R. R. Wu L. L. Mao Y. T. Che F. D. (2024). Fluorescent probes for disease diagnosis. Chem. Rev. 124, 7106–7164. 10.1021/acs.chemrev.3c00776 38760012 PMC11177268

[B44] Wu X. F. An J. M. Shang J. Z. Huh E. Qi S. Lee E. (2020). A molecular approach to rationally constructing specific fluorogenic substrates for the detection of acetylcholinesterase activity in live cells, mice brains and tissues. Chem. Sci. 11, 11285–11292. 10.1039/d0sc04213g 34094370 PMC8162927

[B45] Xing W. T. Yang K. L. Zhu Y. L. Li X. Y. Zhang Y. Guo L. X. (2025). Rational design of a near-infrared fluorescent probe for rapid monitoring of carboxylesterase in live cells and drug-induced liver injury mice. Anal. Chim. Acta 1346, 343782. 10.1016/j.aca.2025.343782 40021330

[B46] Xu N. G. Ge J. Q. Huang K. B. Liu M. Y. Wen Z. Sang S. G. (2025). Activatable proximity-labeled fluorogenic probe for visualizing the role of estrogen in regulating liver carboxylesterase expression. Anal. Chem. 97, 13292–13299. 10.1021/acs.analchem.5c01433 40534217

[B47] Yan Q. Q. Liu H. X. Zhu R. Y. Zhang Z. G. (2024). Contribution of macrophage polarization in bone metabolism: a literature review. Cytokine 184, 156768. 10.1016/j.cyto.2024.156768 39340960

[B48] Yang W. Su G. M. Liu Y. H. (2022). Silencing p62 reduces ox-LDL-induced M1 polarization and inflammation in macrophages by inhibiting mTOR/NF-κB signaling pathways. Eur. J. Inflamm. 20, 1721727X221110348. 10.1177/1721727x221110348

[B49] Yuan Q. Chen L. L. Zhu X. H. Yuan Z. H. Duan Y. T. Yang Y. S. (2020). An imidazo[1,5-α]pyridine-derivated fluorescence sensor for rapid and selective detection of sulfite. Talanta 217, 121087. 10.1016/j.talanta.2020.121087 32498830

[B50] Zhang Y. Y. Huang L. Y. Wang H. L. Meng S. X. Yang F. Liu S. Y. (2025a). Visualization of carboxylesterase 2 regulation in kidney injury via an enzyme-activated fluorescent probe. Sens. Actuat. B-Chem. 429, 137309. 10.1016/j.snb.2025.137309

[B51] Zhang Z. M. Li J. K. Ma M. Shi H. Lu M. J. Liang F. H. (2025b). Near-infrared fluorescence imaging tool with large stokes shift for sensitively detecting carboxylesterase 2 and monitoring its expression in non-alcoholic fatty liver disease. Talanta 285, 127378. 10.1016/j.talanta.2024.127378 39689640

[B52] Zhu S. Y. Chen W. Massion A. Li Y. P. (2024). Cell signaling and transcriptional regulation of osteoblast lineage commitment, differentiation, bone formation, and homeostasis. Cell Discov. 10, 71. 10.1038/s41421-024-00689-6 38956429 PMC11219878

