# Supplementary material for: A fluorescent probe with a vanillin–pyridine–imidazole core structure for carboxylesterase detection in macrophage polarization during bone homeostasis
Source: Front Chem. 2025 Aug 21;13:1666238. doi: 10.3389/fchem.2025.1666238 (PMC12408636; doi:10.3389/fchem.2025.1666238)
Supplement: Supplementary file 1 [file DataSheet1.doc]

**Supporting Information**

**A fluorescent probe with** **vanillin-pyridine-imidazole core-structure for** **carboxylesterase detection in** **macrophage polarization in bone homeostasis**

Hailong Xia^1^, Xianghe Wang^2^, Weichun Huang^1^, Xindong Jiang^1^, Xionggao Han^3,*^, Chaoyue Wang^4,*^

^1^ Orthopedics Department, Dongyang People’s Hospital, Jinhua, 322103, China;

^2^ The First Affiliated Hospital of Wenzhou Medical University, Wenzhou, 325000, China;

^3^ Jinhua Institute of Zhejiang University, Zhejiang University, Jinhua, 321002, China;

^4^ Department of Food Science and Nutrition, Hallym University, Chuncheon, 24252, Korea.

*^*^Corresponding authors. E-mail: xionggao414@zju-jhi.com; Wangchaoyue2023@126.com*

**Supplementary Figures**


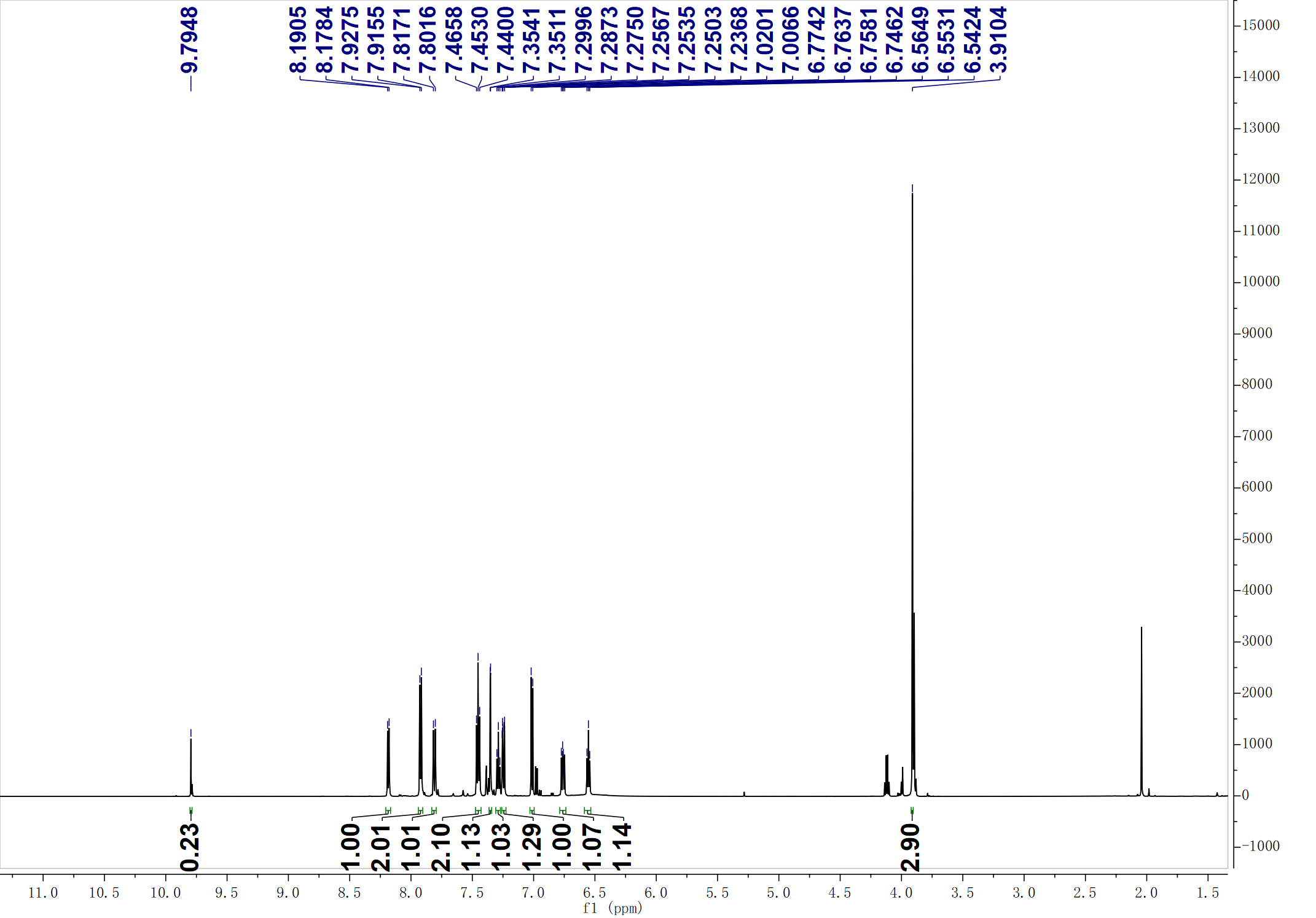


**Figure S1.** ^1^H NMR of the fluorophore **VanPI-OH** (600 MHz, in CDCl_3_).


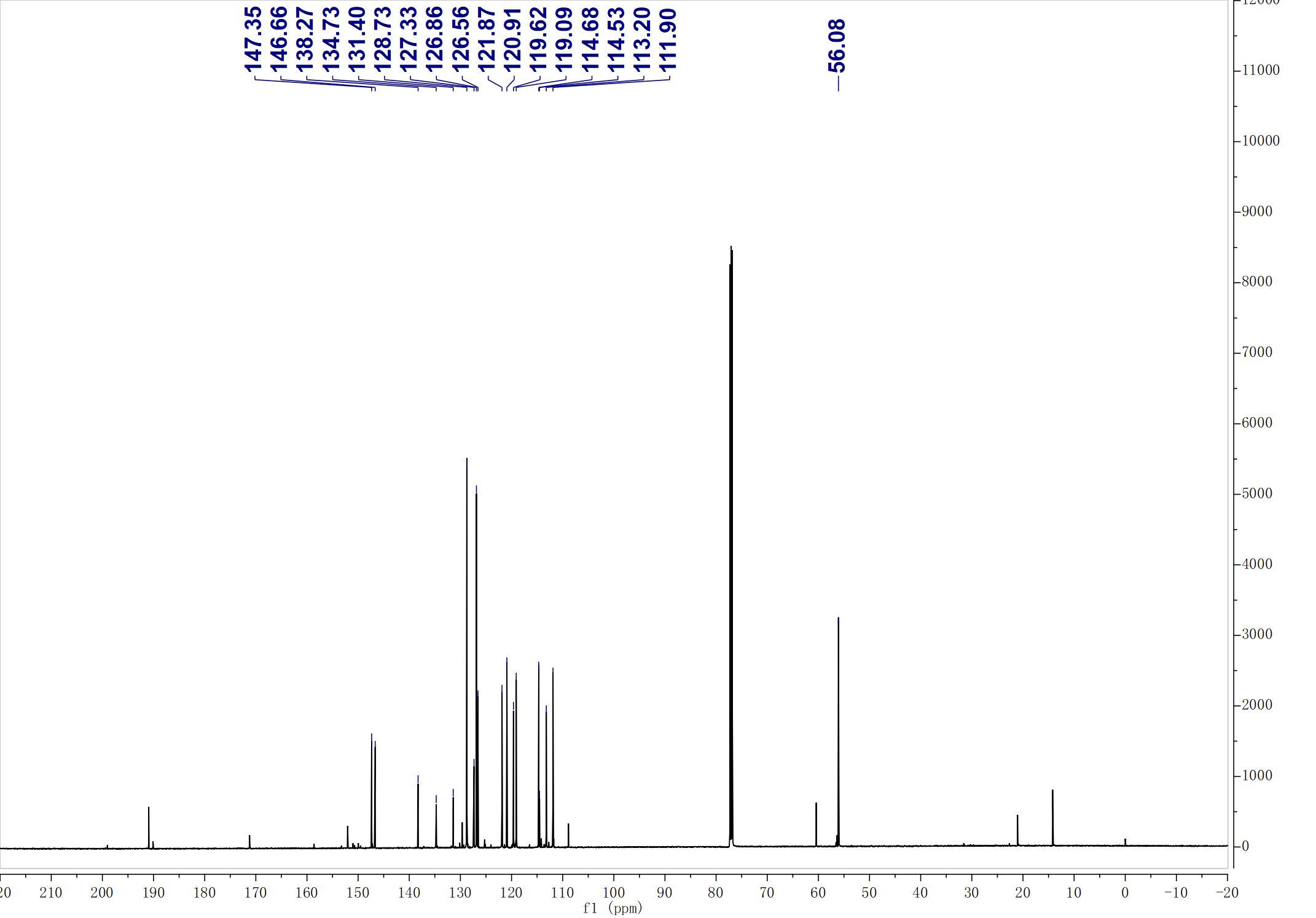


**Figure S2.** ^13^C NMR of the fluorophore **VanPI-OH** (151 MHz, in CDCl_3_).


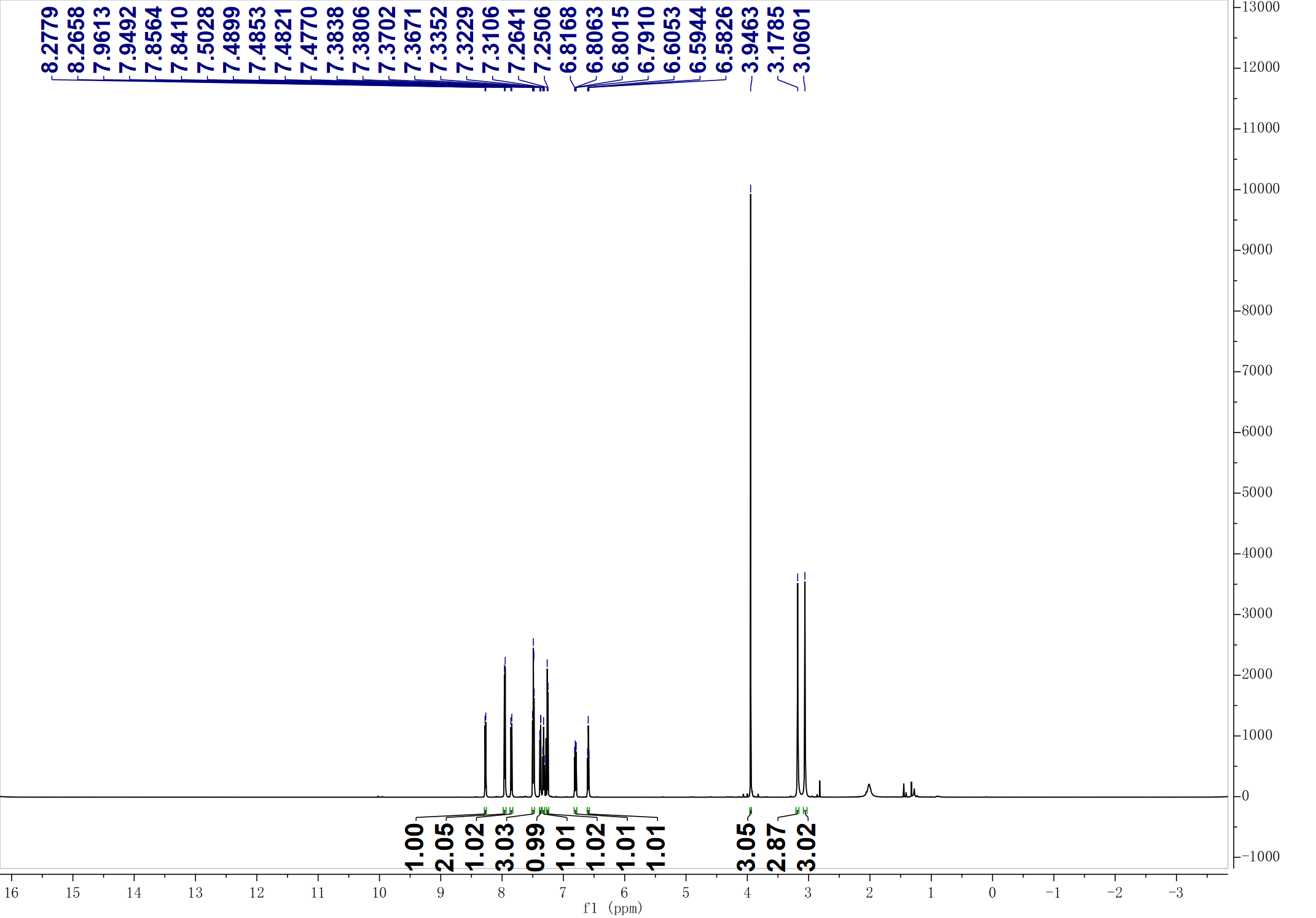


**Figure S3.** ^1^H NMR of the probe **VanPI-CarE** (600 MHz, in CDCl_3_).


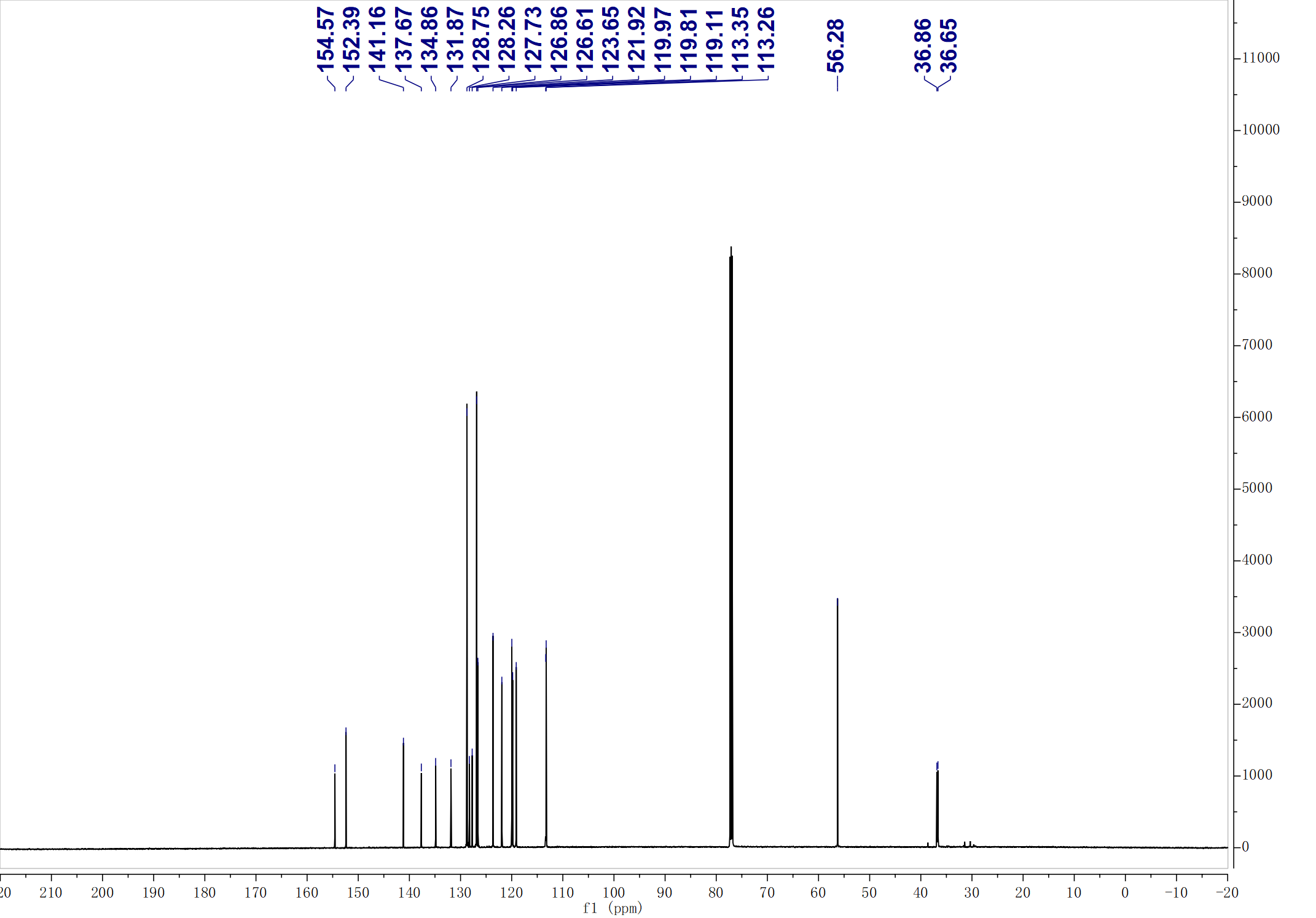


**Figure S4.** ^13^C NMR of the probe **VanPI-CarE** (151 MHz, in CDCl_3_).


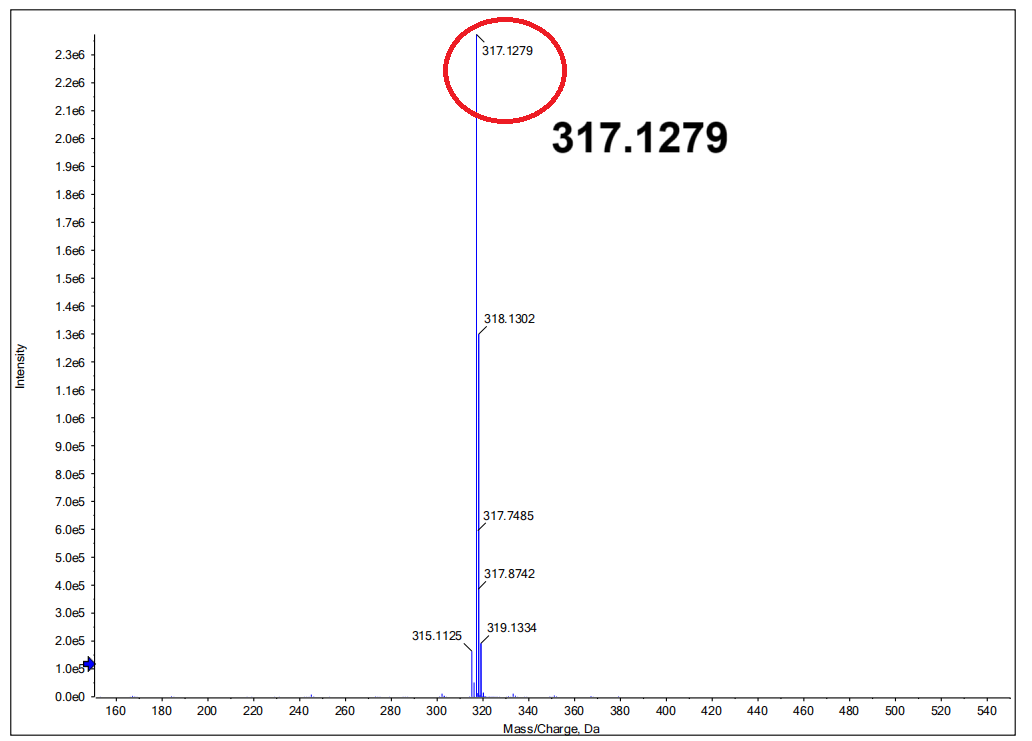


**Figure S5.** HRMS spectrum of the response product **VanPI-OH** in methanol.


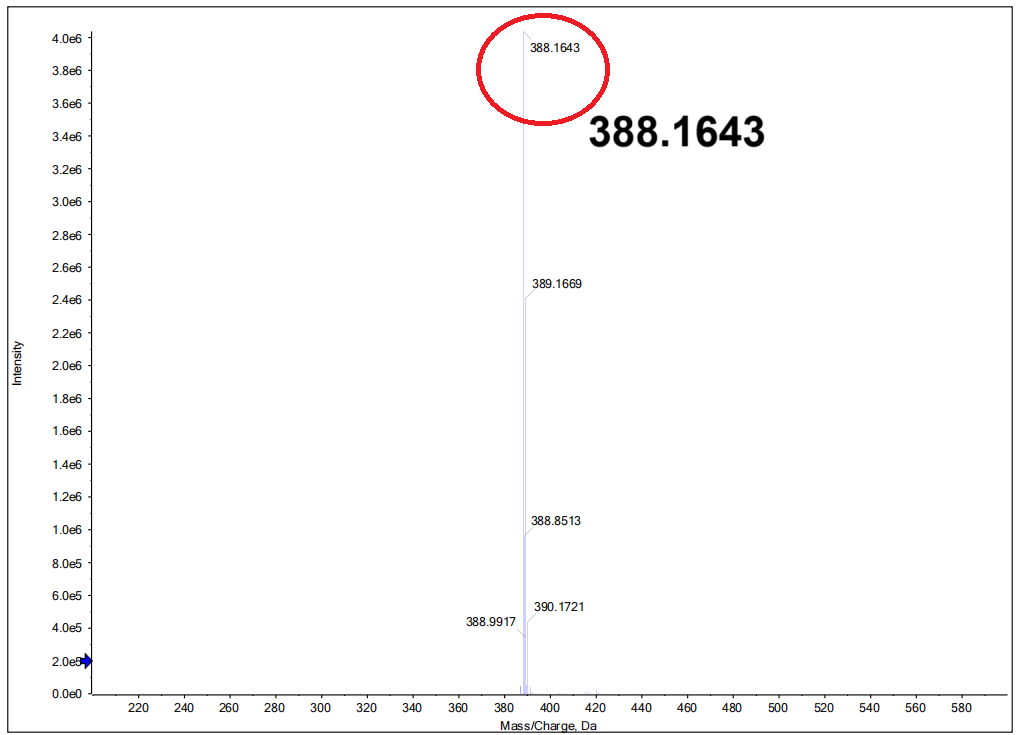


**Figure S6.** HRMS spectrum of the probe **VanPI-CarE** in methanol.


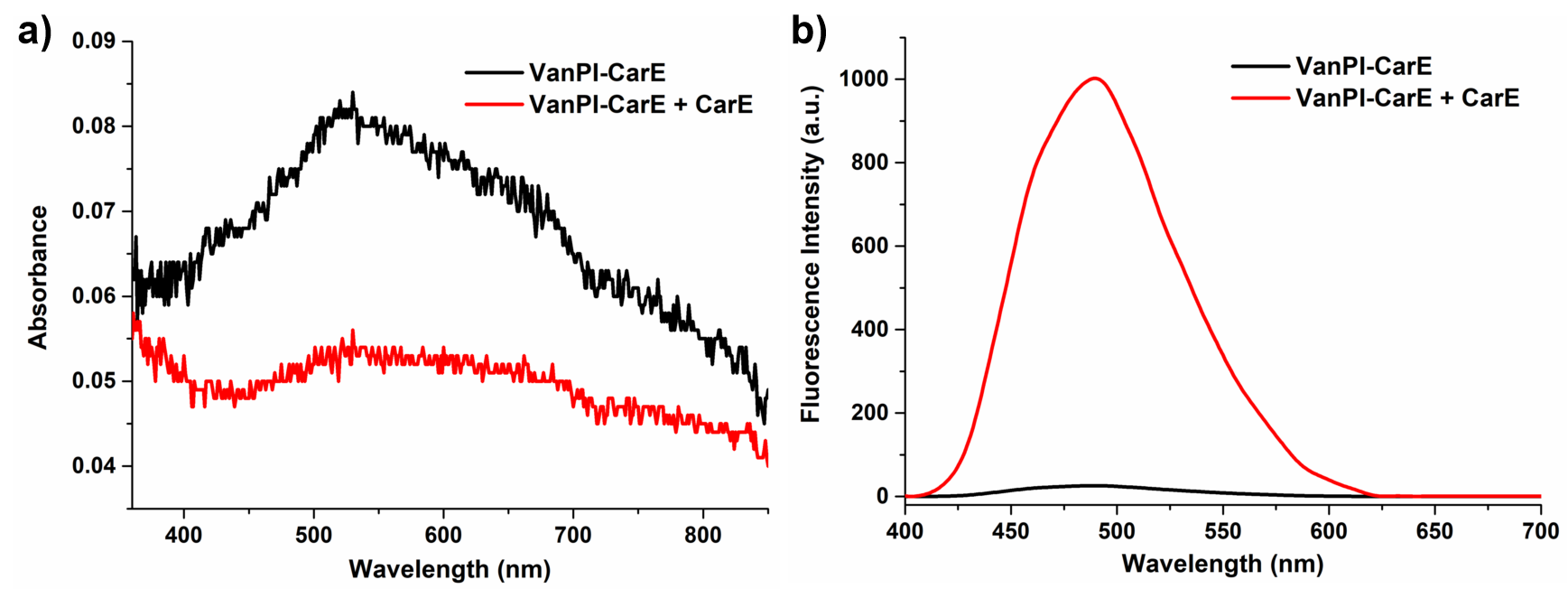


**Figure S7.** (a) Absorbance and (b) fluorescence spectra of the probe **VanPI-CarE** (10 µM) in the absence and presence of CarE (20 U/mL). Conditions: pH 7.4, 37 ℃, 20 min, 5 nm * 5 nm, 600 V, λ_ex_ = 355 nm.


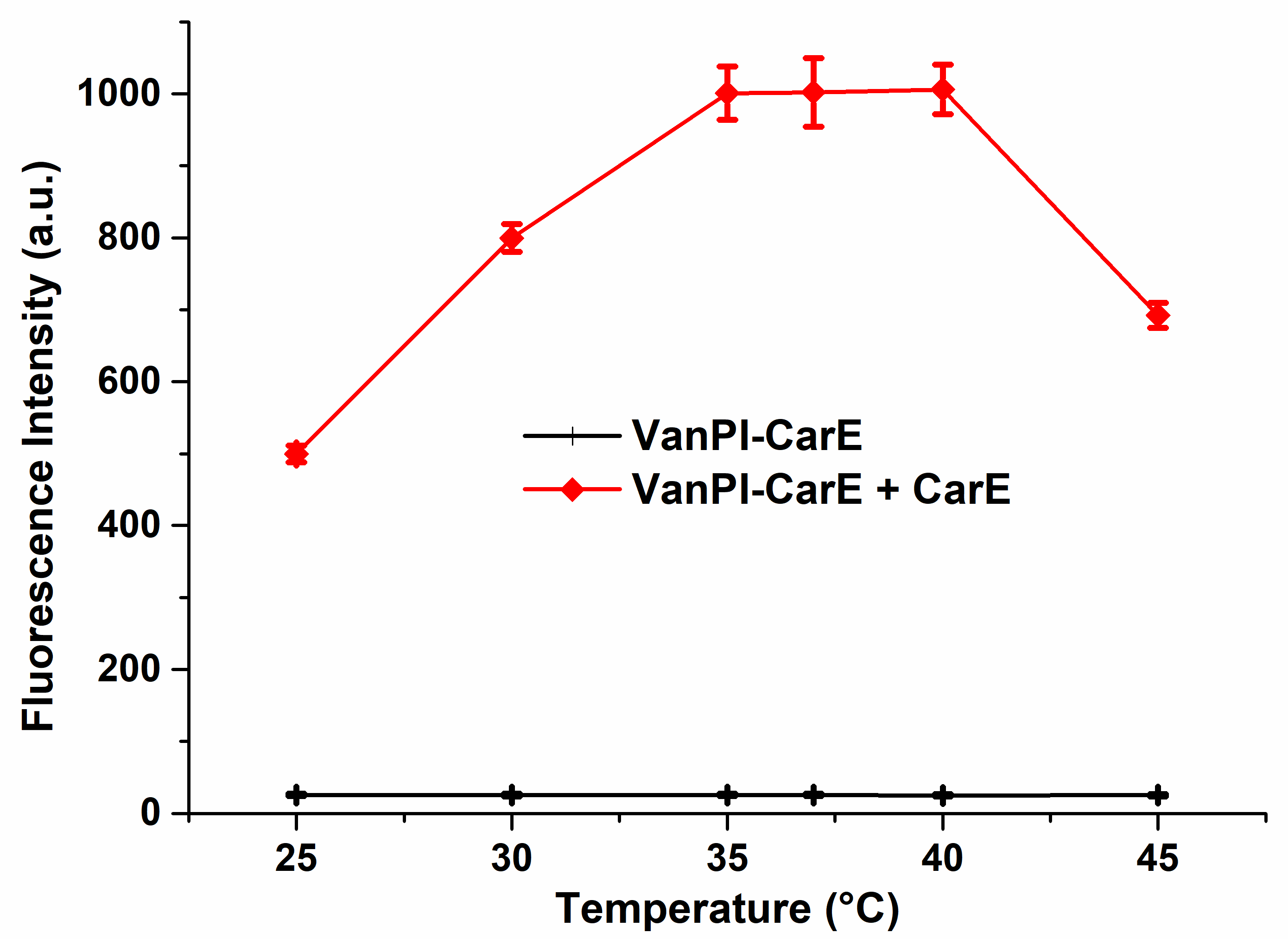


**Figure S8.** Fluorescence intensity at 490 nm of **VanPI-CarE** (10 µM) in the absence and presence of CarE (20 U/mL) in various temperature conditions (25-45 ℃). Conditions: pH 7.4, 20 min, 5 nm * 5 nm, 600 V, λ_ex_ = 355 nm.


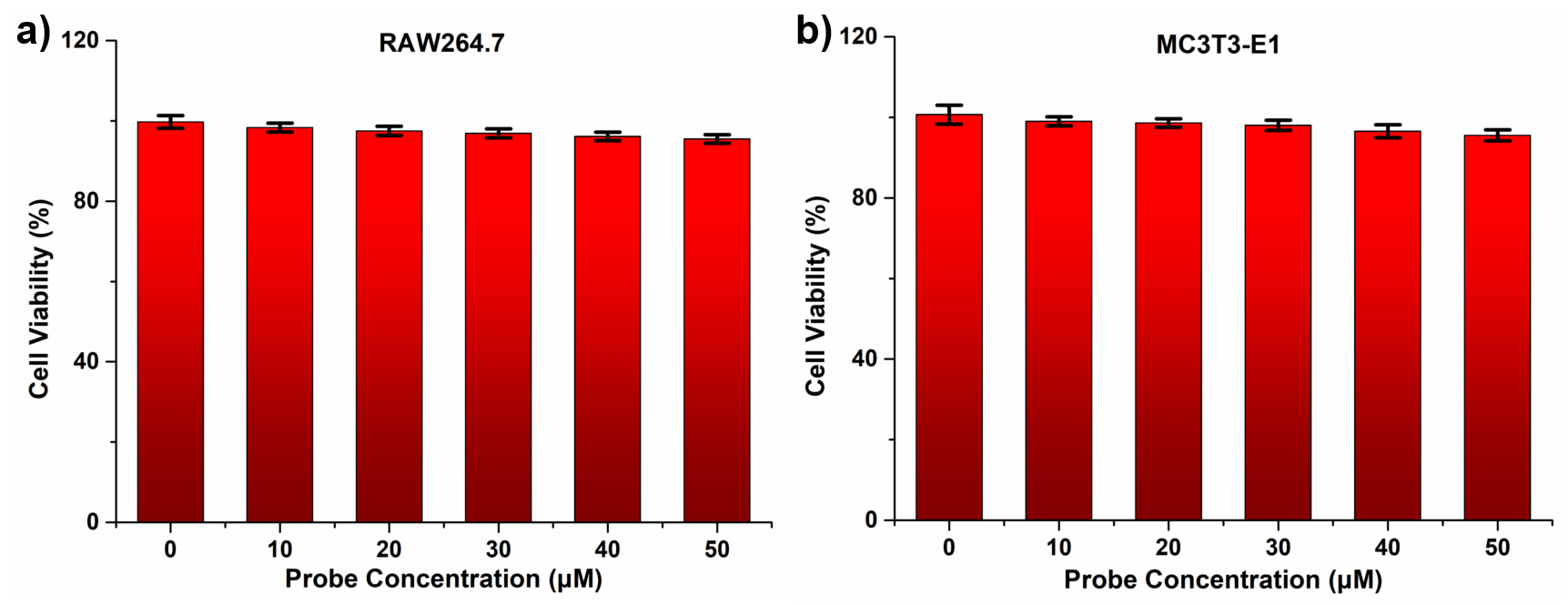


**Figure S9.** Cell viability of (a) RAW264.7 and (b) MC3T3-E1 cells incubated with different concentrations (0-50 μM) of **VanPI-CarE** for 24 h at 37 ℃.
